# A bioartificial dermal regeneration template promotes skin cell proliferation *in vitro* and enhances large skin wound healing *in vivo*

**DOI:** 10.18632/oncotarget.16005

**Published:** 2017-03-08

**Authors:** Peng Chang, Bingyu Guo, Qiang Hui, Xiaoyan Liu, Kai Tao

**Affiliations:** ^1^ Department of Plastic and Reconstructive Surgery, General Hospital of Shenyang Military Area Command, Shenyang, 110840, Liaoning, P.R. China

**Keywords:** bioartificial dermal regeneration template, platelet-rich plasma, acellular collagen sponge, traumatic wound healing, skin cell proliferation

## Abstract

A novel bioartificial dermal regeneration template has been developed using platelet-rich plasma and acellular animal skin collagen sponge for the treatment of larger area and full thickness skin wounds. This platelet-rich plasma-collagen sponge keeps native skin structure and contains huge amounts of growth factors. The effect of this bioartificial dermal regeneration template was tested *in vitro* and *in vivo* via a mimic poor wound healing process by adding collagenase I into cell culture medium or the wound area. The *in vitro* experimental results indicated that the rat skin cells grew faster and produced more collagen in platelet-rich plasma-collagen sponge with collagenase than those treated either with collagen sponge plus collagenase, or collagenase, or control group without treatment. The *in vivo* experiments were performed by large rat skin wounds, 1.5 cm diameter, treated either with collagenase, or collagenase plus collagen sponge, or collagenase plus platelet-rich plasma-collagen sponge. The wound without treatment was used as a control. The wounds treated with collagenase-containing platelet-rich plasma-collagen sponge healed 4 times faster than the untreated wounds, 6 times faster than the collagenase treated wounds, 2.4 times faster than collagenase-containing collagen sponge treated wounds. The immunostaining indicated that the healed tissues in the wound areas treated with collagenase-containing platelet-rich plasma-collagen sponge were composed of collagen type I and collagen III with blood vessels and hair follicles. The results demonstrated that this collagenase-containing platelet-rich plasma-collagen sponge works as a bioartificial dermal regeneration template. The application of this collagenase-containing platelet-rich plasma-collagen sponge promotes the traumatic skin wound healing and permits the reconstitution of the inherent barrier functions of the skin.

## INTRODUCTION

Each year in the United States more than 1.25 million people have burns and 6.5 million have chronic skin ulcers caused by pressure, venous stasis, or diabetes mellitus [1, 2]. Most of these wounds, no matter if from acute injury or chronic wounds are difficult to heal due to the large amount of skin loss. Therefore, wound healing is still a major therapeutic challenge for clinicians, surgeons, and other healthcare professionals.

Diabetes mellitus is a chronic disease that affects the lives of about 16 million people in the United States. The growth of the disease worldwide is especially alarming. Diabetic skin ulcers are a grave complication of diabetes mellitus, occurring with a predicted lifetime risk as high as 25% [3, 4]. Diabetic wounds with additional comorbidities are costly, time intensive, and difficult to heal [5] due to the lack of the necessary growth factors to maintain the healing process [6].

In recent years, platelet-rich plasma (PRP) has been successfully used in many medical fields for treating chronic [6], non-healing wounds [7], open wounds [8], cutaneous wounds [9], soft tissues wounds [10] and bone repair [11] as an autologous blood product. However, only minimal research has been done to assess the impact of PRP treatment as an effective adjunct therapy to wound healing and most of them are case reports rather than original research study. In addition, there are many limitations for PRP application on wound healing because PRP is produced in an autologous or homologous manner and used individually without FDA approval. The first limitation is that some patients have some platelet problems including lower platelet count and lower platelet function so that they cannot use their own PRP for wound healing. The second limitation is that PRP contains many growth factors including transforming growth factor beta 1 (TGF-β1) and highly concentrated PRP may cause scar tissue formation. Whether allogeneic PRP can be used instead of autologous PRP for wound healing and whether PRP mixed collagen will improve severe wound healing are largely unknown.

There is an urgent need to develop an efficiency model for studying the effect of PRP on wound healing, especially on diabetic wound healing. Therefore, the objective of this study is to mimic the diabetic wound healing processes so as to investigate the effect of PRP and collagen on wound healing using collagenase, collagen, and allogeneic PRP to treat a rat's severe skin wound. Our hypothesis is that diabetic wounds produce high concentration of collagenase which inhibits wound healing, while the addition of the mixture of collagen and PRP will decrease the concentration of collagenase and enhance wound healing. To test this hypothesis we first studied the effect of collagenase, collagen and PRP on rat skin cells using an *in vitro* model. Then we determined the effect of collagenase, collagen and PRP on wound healing using an *in vivo* rat model.

## RESULTS

**Figure 1:**
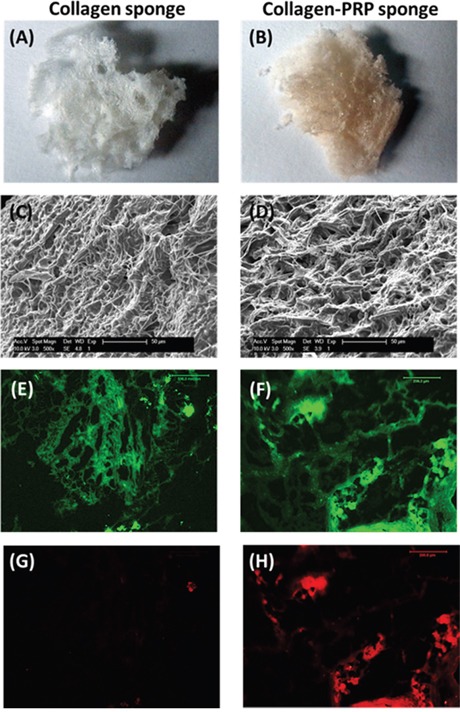
Characterization of the sponges used for wound healing A sponge-like hydrogel has been made either by rat skin collagen only **(A, C, E, G)** or by a mixture of rat skin collagen and rat PRP **(B, D, F, H)**. Scanning electron microscopy images showed the network of collagen fibers under the surface and the spongy internal structure with pores interconnected to support fluid flow **(C, D)**; Immunostaining indicated that collagen type I was positively stained with green fluorescence in both collagen sponges made by collagen only **(E)** and a mixture of collagen and PRP **(F)**. Higher percentage of TGF-β1 was found in collagen and PRP sponge with red fluorescence **(H)** than that in collagen sponge **(G)**.

In order to enhance the severe wound healing, we developed a novel collagen-PRP sponge pad and used it as a bioartificial dermal regeneration template to cover the large wound area. We first extracted collagen from rat skin using the modified protocol and prepared the collagen sponge using rat skin collagen only (collagen sponge, Figure 1A) or mixing skin collagen and PRP of the rats (collagen-PRP sponge, Figure 1B). Gross-view images showed that both collagen sponges had a similar structure (Figures 1A, 1B). SEM images demonstrated that these two kinds of collagen sponges kept collagen fibers in very similar cross-link network (Figures 1C, 1D). However, the pore size and the diameter of collagen fiber in the collagen-PRP sponge (Figure 1D) were larger than those in the collagen sponge (Figure 1C). Immunostaining further indicated that the most collagen in the sponges was collagen type I as evidenced by green fluorescence (Figures 1E, 1F). However, the PRP-containing collagen sponge not only positively stained by collagen type I (Figure 1F), but also positively stained by TGF-β1 (red, Figure 1H). In contrast, very few components in collagen sponge were positively stained by TGF-β1 (red, Figure 1G).

**Figure 2:**
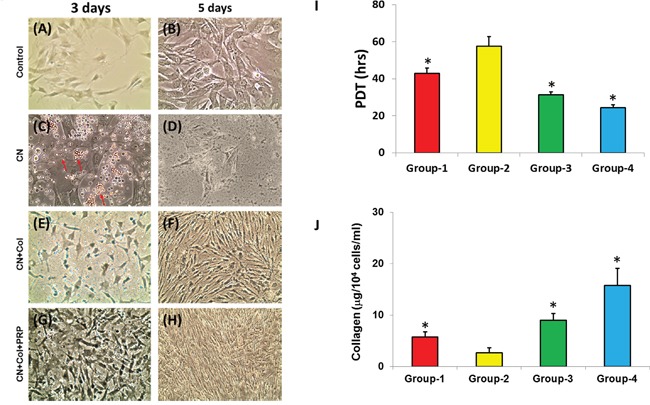
Proliferation and collagen production of rat skin cells cultured in four conditions The cells were cultured in tissue culture plate either with growth medium (control; Group-1) for 3 days **(A)** and 5 days **(B)**; or with growth medium plus collagenase (CN; Group-2) for 3 days **(C)** and 5 days (**D**); or with rat skin collagen sponge in growth medium plus collagenase (CN+Col; Group-3) for 3 days **(E)** and 5 days **(F)**;or with rat skin collagen and PRP sponge in growth medium growth medium plus collagenase (CN+Col+PRP; Group-4) for 3 days **(G)** and 5 days (**H**). The population doubling time (PDT) indicated that the rat skin cells grew much faster in Group-4 than the other three groups **(I)**. The proliferation of rat skin cells cultured under four conditions was in the following order: Group-4 > Group-3 > Group-1 > Group-2 **(I)** (*p<0.05, compared to Group-2). The collagen production in the medium of Group-4 was 2.5 times higher than that of Group-1, 5 times higher than that of Group-2, and 1.5 times higher than that of Group-3 **(J)** (*p<0.05, compared to Group-2).

To understand the cellular and molecular biological mechanisms of wound healing, especially for diabetic wound healing, an *in vitro* cell culture model was used. The proliferation of rat skin cells grown in four different conditions indicated that at day-3 after seeding, more than 90% of rat skin cells in control group cultured with growth medium (10%FBS-DMEM) have attached to the culture plate surface and shown in healthy skin fibroblast shape (Figure 2A). Adding collagenase into culture medium, a mimic diabetic wound condition caused cell apoptosis and more than 70% of the cells in collagenase-containing medium have not attached or died (arrow, Figure 2C). However, the effect of collagenase on rat skin cells was inhibited by using collagen sponge as evidenced by cell shape, cell size, and apoptosis cell numbers shown in Figure 2E. Furthermore, the cells grew much faster in collagen-PRP sponge even with collagenase culture (Figure 2G). After the cells were cultured under the four conditions for 5 days, about 85% of cells in collagenase group have died (Figure 2D) compared to the control group (Figure 2B). Higher density of the cells with healthy morphology was found in collagenase-collagen sponge group (Figure 2F) and collagenase-collagen-PRP sponge group (Figure 2H). The population doubling time (PDT) of the cells grown in collagen-PRP sponge cultured with collagenase-containing medium was much shorter than the other three groups (Figure 2I). The longest PDT was found in the cells grown in the collagenase-containing medium (Group-2; Figure 2I). These results indicated that the proliferation of the rat skin cells was inhibited by collagenase. The order of the proliferation of rat skin cells was Group-4 > Group-3 > Group-1 > Group-2 (Figure 2I). The collagen production in the medium of Group-4 was 2.5 times higher than that of Group-1, 5 times higher than that of Group-2, and 1.5 times higher than that of Group-3 (Figure 2J) (*p<0.05, compared to Group-2).

**Figure 3:**
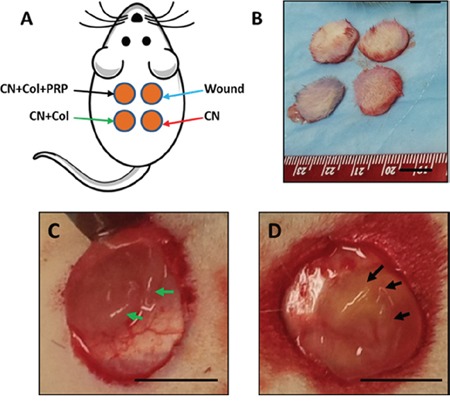
An *in vivo* wound healing model **(A)** The picture shows that four wounds with each 1.5 cm diameter circle were created on the rat back and treated either with collagenase plus collagen-PRP gel (CN+Col+PRP, black arrows); or with collagenase plus collagen sponge (CN+Col, green arrows); or with collagenase (CN, red arrows); or without treatment (Wound, blue arrows). **(B)** Four skin tissue pieces were cut off from rat back of the wound areas. **(C)** A typical wound was covered with CN+Col sponge (green arrows); **(D)** A typical wound was covered by CN+Col+PRP sponge (black arrows). Bars: 1 cm.

The effect of collagen and collagen-PRP sponges on wound healing was also investigated *in vivo* using a rat skin wound model. Full thickness of skin with 1.5 cm diameter circle was cut off as a skin wound from the rat back at four different positions (Figure 3A). The cutoff skin pieces from the wound areas had similar size and shape (Figure 3B). One of the wounds at the upper left side was covered with a collagenase loaded collagen-PRP sponge (CN+Col+PRP; Figures 3A, 3D, black arrows); the lower left wound was treated with collagenase loaded collagen sponge (CN+Col; Figures 3A, 3C, green arrows); the upper right wound was used as a control without treatment (Wound; Figure 3A, blue arrow); the lower right wound was treated with collagenase only (CN; Figure 3A, red arrow).

**Figure 4:**
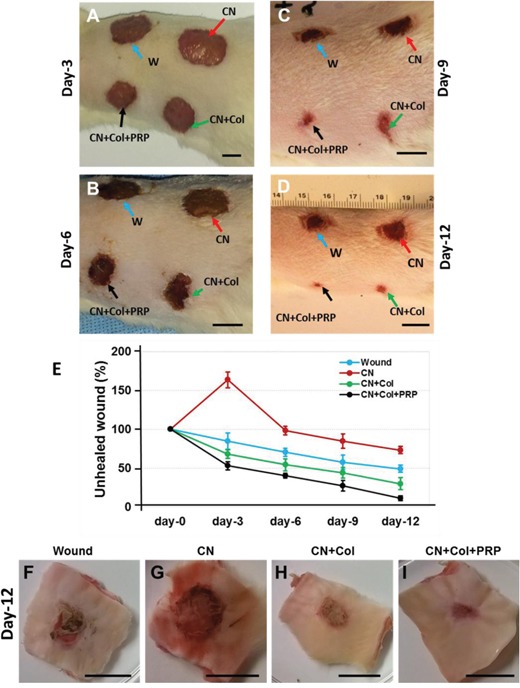
Healing stages of the skin wounds with four different treatments during 12 days **(A)** Healing stage at day-3 post-surgery. **(B)** Healing stage at day-6 post-surgery. **(C)** Healing stage at day-9 post-surgery. **(D, F, G, H, I)**. Healing stage at day-12 post-surgery. **(E)** Semi-quantification of unhealed wound areas at each time point. At day-3 post-surgery, the wound treated with collagenase (CN) was much larger than the other groups (red arrow, **A**) and semi-quantification indicated that the wound area of CN group increased to 150% compared to the wound at day-0 and the wounds without treatment **(E)**. Collagenase inhibited wound healing at all time points (red arrows; **A-D**) and this inhibition effect of collagenase was decreased by collagen sponge (CN+Col, green arrows, **A-D; E, H**) and collagen-PRP sponge (CN+Col+PRP, black arrows, **A-D; E, I**) at all time points. At day-12 post surgery, about 90% wound treated with CN+Col+PRP sponge has been healed **(D, E, I)**, however, large inflammation-like tissues were still found in the wounds without treatment (blue arrow; **D, F**) and treated with CN (red arrow; **D, G**). Bars: 1 cm.

At day-3 post-surgery, the wounds treated with collagenase (CN) was much larger than the other groups (red arrow, Figure 4A), semi-quantification showed that the wound area of CN group on average increased to 150% compared to the original wound size (1.5 cm diameter circle at day-0) (Figure 4E) and the wounds without treatment (blue arrow, Figure 4A). This larger wound area was found at all time points (Figure 4), indicating that the wound healing was inhibited by collagenase. However, the inhibition effect of collagenase was decreased by collagen as evidenced by smaller unhealed wound areas found in all collagen-containing sponges including collagen sponge (CN+Col, green arrows; Figure 4) and collagen-PRP sponge (CN+Col+PRP, black arrows; Figure 4) at all time points. In addition, adding PRP into collagen sponge not only inhibited collagenase effect on wound healing but also enhanced skin wound healing as evidenced by the smaller unhealed wound areas found in CN+Col+PRP sponge treated group than the other three groups at all time points (black arrows; Figure 4). At day-12, wounds treated with CN+Col+PRP has been healed by more than 90% (black arrows, Figures 4D, 4E, 4I). However, large inflammation-like tissue areas were still found in the wounds without treatment (Figures 4D, 4E, 4F) and treated with collagenase (Figures 4D, 4E, 4G).

**Figure 5:**
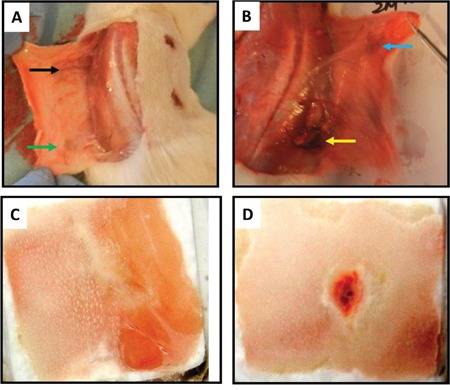
Gross-view images of the inside rat back skin wounds with four different treatments for 12 days **(A)** the inside surface of the wound treated with collagenase loaded collagen-PRP sponge (CN+Col+PRP; black arrow), or the inside surface of the wound treated with collagenase loaded collagen gel (CN+Col; green arrow); **(B)** wound was treated with collagenase (CN; yellow arrow), or without treatment (Wound; blue arrow); **(C)** middle layer of the wounded rat skin cut from inside surface treated with CN+Col+PRP showed the healing has been completed ; and **(D)** middle layer of the wounded rat skin cut from inside surface treated with CN showed scar tissue and unhealed wound area.

There was no unhealed wound area found in the inside skin with CN+Col sponge (green arrow, Figure 5A) and CN+Col+PRP sponge treatments (black arrow, Figure 5A). Although many blood vessels were found in the inside skin of the wound area treated with CN+Col+PRP sponge (black arrow, Figure 5A), the skin color found in the wound areas treated with CN+Col sponge and CN+Col+PRP sponge is normal (Figure 5A). However, the hemorrhage with membrane adhesion was found in the inside skin of the wound area without treatment (blue arrow, Figure 5B), and larger in the wound treated with collagenase (yellow arrow, Figure 5B). A normal skin tissue with normal color and structure was found in the middle layer of the rat skin wound area treated with CN+Col+PRP sponge when the cutting direction of the sections was from inside to the outside (Figure 5C). However, a large scar tissue area was found at the CN treated wound area (Figure 5D).

**Figure 6:**
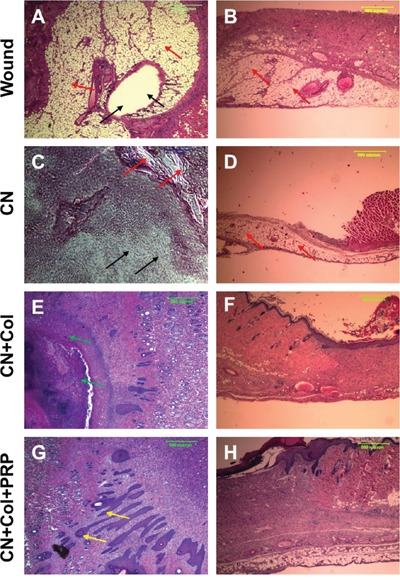
H & E staining of rat skin wound sections with four different treatments for 12 days **(A, C, E, G)** Flat sections of rat skin wound areas; **(B, D, F, H)** Cross sections of rat skin wound areas. The wound without treatment formed fat-like tissue (red arrows; **A, B**) and unhealed wound area was found at day-12 (black arrows; **A**). The wound treated with CN+Col+PRP sponge has been healed completely with normal skin structure **(G, H)**, however, the wound healing was inhibited by collagenase **(C, D)**, large unhealed wound area was still found in CN treated group (black arrows, **C**). The cross section showed that the skin structure of healed tissue was thin without normal dermis layer **(B, D)**. The collagenase inhibition effect was suppressed by collagen sponge **(E, F)** and collagen-PRP sponge **(G, H)**. Although the wound has been healed with CN+Col treatment, some of healed tissues were scar-like tissues (green arrows, **E**). Many hair follicles were found in the healed tissues of the wounds treated with CN+Col+PRP sponge (yellow arrow, **G**).

Histology analysis showed large unhealed wound areas (black arrows) presented in the wounds without treatment (Figures 6A, 6B) and treated with collagenase (Figures 6C, 6D). The wounds with CN treatment (Figures 6C, 6D) or without treatment (Figures 6A, 6B) were filled by fat-like tissues (red arrows, Figure 6). However, the wounds healed much faster with CN+Col sponge treatment (Figures 6E, 6F) and CN+Col+PRP sponge treatment (Figures 6G, 6H). Although the wound has been healed with CN+Col treatment, some of healed tissues were scar-like tissues (green arrows, Figure 6E). Many hair follicles were found in the healed tissues of the wounds treated with CN+Col+PRP sponge (yellow arrows, Figure 6G). The cross-sections indicated healing speed of the wounds with four different treatments by skin thickness and structure. A full thickness skin with three layers was found in healed wound area treated with CN+Col+PRP (Figure 6H). The healed tissue was 50% thinner than normal skin found in CN+Col treated group (Figure 6F), indicating that the wound was not healed completely (Figure 6F). There was no normal skin structure found in new formed tissues of CN treated group and without treatment group (Figures 6B, 6D).

**Figure 7:**
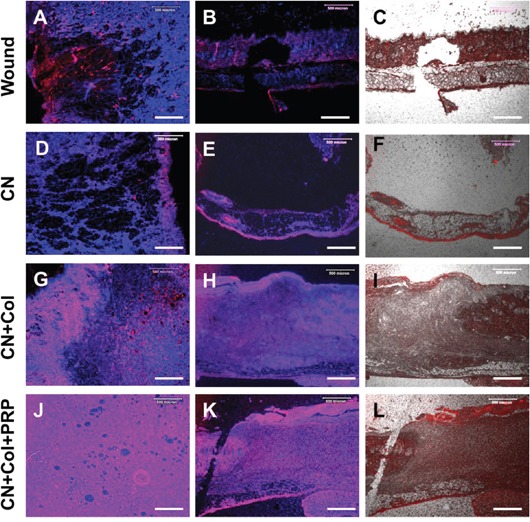
Immunostaining on collagen I expression of rat skin wound sections with four different treatments for 12 days **(A, D, G, J)** Flat sections of rat skin wound areas; **(B, C, E, F, H, I, K, L)** Cross sections of rat skin wound areas. The collagen type I was positively stained by red and the nuclei were positively stained by H33342 (blue). The merged areas (pink) indicated that both collagen I and nuclei were expressed in the tissues. Although the wound area without treatment was filled by some new tissues, about only 30% of the tissues were positively stained by collagen I (red/pink, **A, B, C**). Similarly, less than 15% of the healed tissues in CN group expressed collagen I (red/pink, **D, E, F**). However, more than 90% of the tissues in the wound area treated with CN+Col+PRP sponge were positively stained by collagen **(J, K, L)**, and about 80% of the healed tissue at the wound area treated with CN+Col sponge expressed collagen I **(G, H, I)**. Bars: 500 μm.

**Figure 8:**
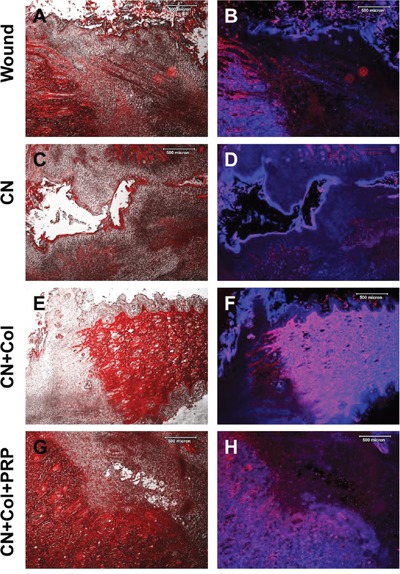
Immunostaining on collagen III expression of rat skin wound sections with four different treatments for 12 days **(A, C, E, G)** Merged images of phase-contrast (black & white) and immunostaining on collagen III (red) of the rat skin wound areas; **(B, D, F, H)** Merged images of immunostaining on collagen III (red) and H33342 staining on nuclei (blue) of rat skin wound areas. Collagen III was strongly expressed in CN+Col group (more than 75%, red, **E**; pink, **F**) and CN+Col+PRP group (about 50%, red, **G**; pink, **H**). However, collagen III was weakly expressed in CN group (less than 20%, red, **C**; pink, **D**) and wound without treatment group (less than 40%, red, **A**; pink, **B**). Bars: 500 μm.

Immunostaining results indicated that the healed tissues in the wound area treated with CN+Col+PRP sponge were more than 90% stained positively with collagen I (Figures 7J, 7K, 7L). Similarly, the tissues formed in the wound area treated with CN+Col sponge were about 80% stained positively by collagen I (Figures 7G, 7H, 7I). However, about 30% of tissues at wound area without treatment expressed collagen I (Figures 7A, 7B, 7C) and less than 15% of the tissues presented collagen I at the wound area treated with CN (Figures 7D, 7E, 7F). Collagen III was also positively stained in all four groups (Figure 8), indicating the new tissue formation at the wound areas. However, collagen III was weakly stained (less than 20%) in the tissue of the wound areas treated with collagenase (Figures 8C, 8D). In addition, more than 75% of the wound areas treated with CN+Col were positively stained by collagen III (Figure 8E, 8F), about 50% of the wound areas treated with CN+Col+PRP (Figures 8G, 8H), and less than 40% of the wound areas without treatment (Figures 8A, 8B) expressed collagen III.

**Figure 9:**
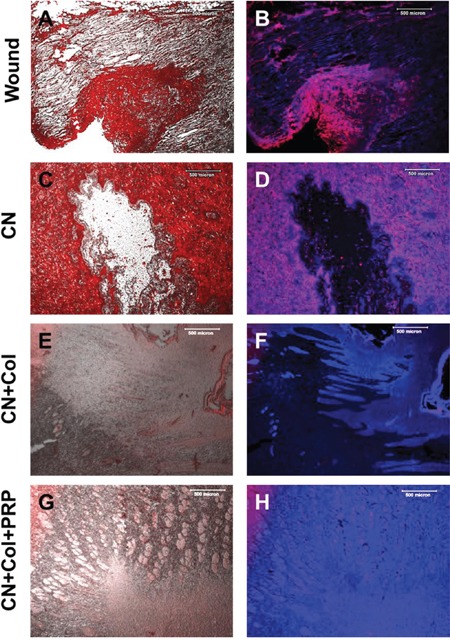
Immunostaining on MMP-3 expression of rat skin wound sections with four different treatments for 12 days **(A, C, E, G)** Merged images of phase-contrast (black & white) and immunostaining on MMP-3 (red) of the rat skin wound areas; **(B, D, F, H)** Merged images of immunostaining on MMP-3 (red) and H33342 staining on nuclei (blue) of rat skin wound areas. The merged areas (pink) indicated that both collagen I and nuclei were expressed in these tissues. MMP-3 was strongly stained in the wound areas treated with collagenase (more than 90%, red, **C**; pink, **D**) and the wounds without treatment (about 50%, red, **A**; pink, **B**). However, MMP-3 was weakly expressed in CN+Col group **(E, F)** and in CN+Col+PRP group **(G, H)**. Bars: 500 μm.

In addition, more than 90% of the tissues at the wound area treated with collagenase were positively stained by matrix metalloproteinases-3 (MMP-3) (Figures 9C, 9D). About 50% of the wound areas without treatment also expressed MMP-3 (Figures 9A, 9B). However, very weak expression of MMP-3 was found in CN+Col sponge treated group (Figures 9E, 9F) and CN+Col+PRP treated group (Figures 9G, 9H).

**Figure 10:**
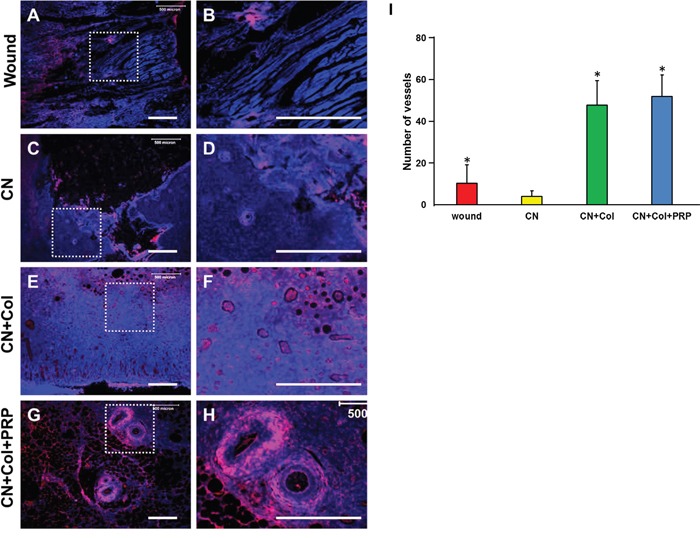
Immunostaining on the expression of von Willebrand factor (vWF) in the rat skin wound sections with four different treatments for 12 days **(A, B)** Wound without treatment. **(C, D)** Wound treated with CN. **(E, F)** Wound treated with CN+Col. **(G, H)** Wound treated with CN+Col+PRP. The images of B, D, F, and H are enlarged images of A, C, E, and G, respectively. **(I)** Number of blood vessels in healed tissues of each group. Blood vessels in the wound areas treated with two collagen sponges were 10 times more than that in CN treated wounds and four times more than that in the wounds without treatment (*p < 0.05, compared to CN group). There was no significant difference between the numbers of blood vessels in two collagen sponge groups, but larger size of the blood vessels was found in the wound area treated with CN+Col+PRP sponge. Bars: 500 μm.

Finally, the expression of Von Willebrand factor (vWF) was increased in both Col-containing sponges (Figures 10E-10I), however, vWF expression was more extended in CN+Col+PRP group (Figures 10G, 10H, 10I). The blood vessels in the wound areas treated with two collagen-containing sponges were 10 times more than CN treated groups (Figures 10E, 10F, 10I). In addition, the vWF level was decreased in CN treated group (Figures 10C, 10D) when compared to the wounds without treatment (Figures 10A, 10B). There was no significant difference between the numbers of blood vessels in two collagen sponge groups, but larger size of the blood vessels was found in the wound area treated with CN+Col+PRP group (Figures 10G, 10H).

## DISCUSSION

Skin is the largest organ of human body, which acting as a barrier with immunologic, sensorial and protective functions against the external environment [12]. Once skin is injured, micro-organisms that are normally sequestered at the skin surface obtained access to the underlying tissues. The longer a wound exists, the greater the potential for infection. The loss of integrity of skin by traumatic experiences such as burns and ulcers may result in considerable disability or ultimately death [13]. How to enhance the skin wound healing is still a big challenge for both clinical doctors and scientific researchers.

Several approaches are now used to treat skin lesions and damages including the application of autografts, allografts, tissue-engineered substitutes, wound dressings and nanofiber scaffolds [12]. Even though proven clinically effective, these methods still have some limitations. Autograft is the best material for the treatment of large skin damage; however, some patients do not have enough donor sites for autograft preparation. Besides the source limitation, a second wound is created in order to gain a skin graft and the donor site wounds are often more painful than the skin graft wound due to the exposure of sensory nerve endings [14]. A cadaver allograft is also the preferred material for temporary closure of excised wounds in patients with extensive, life-threatening burns and inadequate donor sites [15]. However, the allograft has the risk of immunoreactions and disease transmission. Advanced approaches based on tissue-engineered skin substitutes and dermal analogs have offered a permanent, viable and effective substitute to explain the drawbacks of skin regeneration and repair by combining growth factors, cells, and biomaterials and advanced biomanufacturing methods [12].

In the current study, we have developed a novel approach for large skin wound treatment using PRP-containing rat skin collagen sponge as a bioartificial dermal regeneration template. Immunostaining showed that this PRP-collagen sponge contained growth factors and bioactive reagents, such as TGF-β1 and collagen type I (Figures 1F, 1H). The population doubling time (PDT) indicated that this PRP-collagen sponge promoted the proliferation of rat skin cells *in vitro* (Figure 2I) and enhanced the skin wound healing *in vivo* (Figure 4).

Wound healing is a complex multi-cellular process involving fibroblasts, keratinocytes, and endothelial cells as well as inflammatory cells [16]. Collagen is a keystone of skin formation and repair, playing a crucial role in the maintenance of skin tensility and elasticity. In healthy human skin, type I and III collagen have relatively substantial roles during collagen formation, comprising of 80-85% and 10-15% in human skin, respectively [17]. It has been reported that collagen and collagen-derived fragments control many cellular functions, including cell shape and differentiation [18], migration [19], and synthesis of many proteins [20]. Our *in vitro* results showed that rat skin cells grew much faster in collagen-containing sponges than control group without collagen-containing sponge (Figure 2). These results were further confirmed by *in vivo* experiments. The wounds treated with collagen-containing sponges healed much quicker than the wounds treated without collagen-containing sponges (Figure 4). The immunostaining results demonstrated that expression of collagen I and III increased in the wounds treated with collagen-containing sponges (Figures 7, 8).

Matrix metalloproteinases (MMPs) also play an important role in wound healing [21]. Previous studies have shown that delayed healing is characterized by an increase in MMPs [22] and a reduction in some growth factors, such as TGF-β [23]. It has been demonstrated that the excess protease activity in the wound can lead to a chronic non-healing wound. In the present study, we mimic poor wound healing process by adding collagenase I (MMP1) into the wound area. Our results showed that regulation of MMP levels in wounds could lead to improved wound healing. More than 73% of unhealed wound areas were found in collagenase treated group at day-12 post-surgery (Figures 4 and 5). The expression of MMP-3 increased in collagenase treated group (Figure 8). There was no blood vessel formation found in collagenase treated wound tissues (Figure 10). However, the collagenase effect was inhibited by collagen-containing sponges no matter collagen only or collagen mixed with PRP together. The wounds treated with collagenase plus collagen-containing sponges healed much faster than collagenase treated group. The MMP-3 expression decreased in collagen-containing sponge treated groups. Some blood vessels were formed in collagen-containing sponge treated wounds.

Most skin wounds cause leakage of blood from damaged blood vessels. After the wound has been inflicted, homeostasis begins. A clot is formed by platelets and then serves as a temporary shield protecting the denuded wound tissues and provides a provisional matrix over and through which cells can migrate during the repair process. In the present study, we mixed PRP and collagen together to develop a PRP-collagen sponge and used as a novel bioartificial skin substitute to treat large skin wound. It has been reported that PRP contains a high level of platelets and a full complement of clotting and growth factors [24] [25]. PRP functions as a tissue sealant and drug delivery system, with the platelets initiating wound repair by releasing locally acting growth factors via a-granules degranulation [26]. In addition, PRP serves as a growth factor agonist [27] and releases growth factors when PRP is activated by collagen. Our results indicated that this PRP-collagen sponge not only stopped blood but also enhanced wound healing.

In this study, we used allogeneic PRP instead of autologous PRP to treat skin wound. Although autologous PRP has been widely used to treat many wounds, the efficacy of PRP treatment is controversial due to inconsistent results from the human clinical trials. One of the reasons is that the components of the PRP are largely different because the PRP is prepared individually. The findings from this study indicated that allogeneic PRP can be used to instead of autologous PRP to treat large skin wound and showed that the allogeneic PRP has more advantages than autologous PRP because the preparation protocol and formula are standard and no source limitation.

Cell proliferation plays an important role in the second wound healing stage. For the granulation tissue formation, the blood vessels developed a new network to replace the damaged ones. When blood cannot properly reach the affected area, it becomes malnourished and low in oxygen, thereby stalling the wound healing phases. In our experiments, we added PRP into collagen sponge to enhance the angiogenesis by releasing vascular endothelial growth factor (VEGF) and platelet-derived growth factor (PDGF) from PRP. Our results indicated that many blood vessels have formed in the wound areas treated with collagenase in collagen-PRP sponge (Figures 10G, 10H), and collagenase in collagen sponge (Figures 10E, 10F). However, very few blood vessels were found in collagenase treated (diabetic-like) wounds (Figures 10C, 10D). It has been reported that vWF was expressed weakly in diabetic animals [28].

The color of the granulation tissue is an indicator of the health of the wound. A reddish or pinkish color found in collage-PRP sponge treated wound indicated that the wound was healthy and healed well (Figures 4I, 5A). However, a darker tissue found in collagenase treated wound indicated that the wound was infected or inadequate blood was delivered to the wound bed (Figures 4G, 5B).

The acellular animal collagen sheets have been successfully used as bioartificial dermal regeneration templates (DRT) for the reconstruction of traumatic wounds [29]. The previous studies have demonstrated that using DRT to treat traumatic wounds has several potential benefits including coverage of exposed nerves, blood vessels and/or bone; decreasing or eliminating the need for more morbid procedures such as tissue transfers; promotion of a well-vascularized bed in at-risk or hypovascular wounds; lessening the need for shortening of amputated extremities by providing more stable soft tissue coverage and durability and contouring of soft tissue defects to improve cosmesis, function and/or comfort with prosthetic wear [30] [31]. However, the DRT used in the clinics currently is a porous matrix of cross-linked bovine tendon collagen and shark glycosaminoglycan. In the present study, a novel DRT has been developed by an acellular animal skin collagen sponge with PRP and collagenase for traumatic skin wound healing. Although the treatment efficacy of our PRP-Collagen sponge and commercial DRT made by bovine tendon collagen and shark glycosaminoglycan on skin wound healing has not been compared, it is believed that the ideal acellular matrix is one that most closely approximates the structure and function of the native extracellular matrix (ECM) it is replacing. The acellular skin collagen sponge has more beneficial effects than the acellular tendon collagen sheet for skin wound healing. In addition, PRP-containing DRT has much more beneficial effects on wound healing than the DRT without PRP.

There are several limitations to this study. The first one is that *in vivo* testing was just investigated for 12 days. The long term effect of our collagen-PRP sponge on traumatic wound healing should be studied in the future. The second one is that we just investigated four different treatments including wound without treatment (Wound), wound treated with collagenase (CN), wound treated with collagenase and collagen sponge (CN+Col), and wound treated with collagenase plus collagen-PRP sponge (CN+Col+PRP) on large skin wounds were performed. We didn't study the effect of PRP treatment (PRP), PRP-collagen sponge treatment (PRP+Col), and collagen sponge (Col) on skin wound healing. In the future study, these three additional groups will also be investigated. In this study, one collagenase concentration was investigated. In order to find an effective therapy concentration of collagenase for skin traumatic wound healing, different concentrations of collagenase will be added into the wounds at the different healing stages.

### Conclusion

In this study, we developed a novel PRP-containing, acellular animal skin collagen sponge for the treatment of larger area and full thickness skin wounds. This collagen-PRP sponge keeps native skin structure and contains huge amounts of growth factors. Application of the collagen-PRP sponge promotes the proliferation and collagen production of rat skin cells *in vitro* and enhances traumatic and diabetic skin wound healing *in vivo*. This collagen-PRP sponge works as a bioartificial dermal regeneration template to permit the reconstitution of the inherent barrier functions of the skin. The treatment of collagen-PRP sponge with appropriate concentrations of collagenase provides an efficient approach for severe wound healing.

## MATERIALS AND METHODS

### Animals

Ten-week-old Sprague-Dawley female rats, weighing 250-300 g, were used in this study. All animals were treated according to institutional guideline for laboratory animal treatment and care. All experiments were approved by the Animal Research Ethics Committee of General Hospital of Shenyang Military Area, China.

### Extraction of collagen from rat skin

The collagen was extracted from rat skin according to the published protocol [32] and described as the follows. After rats were sacrificed, rat skin tissues were obtained immediately by dissection. The hair and fatty tissues were removed, and then the skin tissues were immersed into liquid nitrogen for 5 min and grounded into powder. The skin powder was treated with 0.5% trypsin/PBS solution (100 mg/ml) at 37°C under vigorous agitation for 24 hours and the trypsin was changed every 6 hours. Then the skin powder was washed with PBS three times (30 min each time) and digested at 37°C with a nuclease solution (50U/ml DNase and 1U/ml RNase in 10 mM Tris-HCl, pH 7.5) for 12 hours. The digested powder was treated with 1% Triton X-100 for 24 hours at room temperature, washed 6 times with PBS, 8 hours each time, and stored at −80°C for subsequent cell culture or wound healing use.

### Preparation of collagen sponge

Collagen powder (0.5 g) obtained from above treatment was dissolved with 10 ml of 3% acetic acid (HAc, 0.5M) to make a 5% collagen-HAc solution. The collagen gel was made by adding the equivalent of sodium hydroxide (1 ml of 5 M NaOH) to 10 ml of 5% collagen-HAc solution according to the published protocol [32]. The collagen sponge was obtained by the dryness of the collagen gel using a vacuum freeze dryer.

### Preparation of collagen-PRP sponge

After rats were sedated, whole blood was withdrawn from the hearts and mixed each 9 ml of fresh blood with 1 ml of 3.8% sodium citrate. The plasma, leukocytes and red blood cells in sodium citrate treated whole blood (total 10 ml) were separated by a centrifuge at 500g for 10 min. The platelets and leukocytes in each part were counted by an automatic hematology analyzer (Cell-DYN Emerald; Abbott Diagnostics, Abbott Park, Illinois). The PRP use for the following experiments with the concentration of 5 × 10^8^ platelets/ml was prepared according to the published method and activated by 22 mM CaCl_2_ before use [33]. The collagen-PRP sponge was obtained from a collagen gel which was made by mixing 1 ml of 5% collagen-HAc solution with 0.1 ml of 5M of NaOH and 1 ml of PRP and dried by a vacuum freeze dryer.

### Scanning electron microscopy (SEM) of collagen sponge and collagen-PRP sponge

The sponge samples were dried by liquid nitrogen and sputter coated with gold/palladium, then examined under a JEOL (Tokyo, Japan) SEM with an accelerating voltage of 10.0 kV.

### Immunostaining on collagen sponge and collagen-PRP sponge

Both the collagen sponge and collagen-PRP sponge were characterized by immunostaining on collagen type I and TGF-β1 according to the published protocol [32]. The sponge samples were placed in frozen section medium (Neg 50; Richard-Allan Scientific; Kalamazoo, MI) and solidified completely with liquid nitrogen cold 2-methylbutane. The sponge blocks were cut into 10 μm thick sections and dried overnight at room temperature. The sections were rinsed 3 times with PBS, fixed with 4% paraformaldehyde for 30 min, and further washed with PBS three more times. The sections were coated with 5% goat serum for 30 min at room temperature in a humid chamber. The serum was carefully removed by aspiration, then mouse anti-collagen type I antibody (1:350; Santa Cruz Biotechnology, Inc., Santa Cruz, CA) or mouse anti-TGF-β1 antibody (1:350; Santa Cruz Biotechnology, Inc., Santa Cruz, CA) were applied, the sections were incubated with the antibodies at room temperature for 2 hrs. The sponge sections were washed three times with PBS, and then reacted with FITC-conjugated goat anti-mouse IgG (1:500; Santa Cruz Biotechnology) for collagen type I and Cy3-conjugated goat anti-mouse IgG for TGF-β1 at room temperature for 1 hr. The sections were washed three times with PBS, and then the positively stained collagen type I (green) and TGF-β1 (red) were checked under a microscope.

### *In vitro* effect of PRP-Col sponge on rat skin cells

Rat dermal fibroblasts were obtained from ScienCell (Cat. #R2300, ScienCell, Carlsbad, CA) and cultured in growth medium (10% FBS-DMEM) at 37°C with 5% CO_2_ for one week.

The cells at passage 1 were seeded in 6-well plate at the density of 5 × 10^4^/well and cultured in four different conditions. Group 1: The cells were cultured in 6-well plate with 2 ml of growth medium in each well (Control); Group 2: The cells were cultured in 6-well plate with 1.9 ml of growth medium and 0.1 ml of 3 mg/ml collagenase type I (100U) (Collagenase); Group 3: Before seeding the cells, the plate was coated with collagen gel by adding 1 ml of 5% collagen-HAc and 0.1 ml of 5M of NaOH and dried by a vacuum freeze dryer. Then, the plate with collagen sponge was put under UV light in a biological safety cabinet overnight. The collagen sponge was washed with PBS three times, then 75% ethanol for three time, and finally washed with PBS for another three times. The cells were seeded on this collagen sponge and cultured with 1.9 ml of growth medium and 0.1 ml of 3 mg/ml collagenase type I (Collagenase+ Collagen); Group 4: Before seeding the cells, the plate was coated with collagen-PRP gel by adding 0.5 ml of 5% collagen-HAc with 50 μl of 5M of NaOH and 0.5 ml of PRP (5 × 10^8^ platelet/ml) with 10 μl of 2.2 M CaCl_2_, and dried by a vacuum freeze dryer. Then, the collagen-PRP sponge coated plate was treated with the same procedures used for collagen sponge. The cells were seeded in this collagen-PRP sponge and cultured with 1.9 ml of growth medium and 0.1 ml of 3 mg/ml collagenase type I (Collagenase+ Collagen + PRP). The proliferation of rat skin cells grown in these four different conditions was studied by cell morphology observed at day-3 and day-5 post-seeding and population doubling time (PDT) determined at the confluence stage. The PDT is calculated by dividing the total culture time by the number of generations.

Finally, the total collagen production in the medium of rat skin cells cultured under four different conditions was determined according to the published protocol [34] using a Sircol collagen assay kit (Biodye Science, Biocolor Ltd., Carrickfergus, Northern Ireland, UK).

### *In vivo* skin wound healing

Eight female Sprague-Dawley rats (10 weeks old; 250–300g) were used to test the effects of collagenase, collagen and PRP on diabetic-like wound healing *in vivo*. Rats were housed individually on a 12 hrs:12 hrs light–dark cycle and were cared for in accordance with the Guide to the Care and Use of Experimental Animals.

For *in vivo* wound healing experiments, the collagen sponge was made by adding 1 ml of 5% collagen-HAc solution to a 12-well plate and adjusting its pH to 7.0 with 100 μl of 5M sodium hydroxide (NaOH). In addition, collagen-PRP sponge was made by adding 0.5 ml of 5% collagen-HAc solution and 50 μl of 5M NaOH to 0.5 ml of PRP in a 12-well plate. Both kinds of sponges were dried by a vacuum freeze dryer and washed with PBS for three times, then 75% ethanol for three times, finally, PBS for another three times before use. The surgeries were performed on the rats under general anesthesia using isoflurane. Two circular skin defects with a diameter of 1.5 cm were created in two distinct places on each side of the rat back. One wound was without treatment (Wound only), one wound was filled with 1 ml of 3 mg/ml collagenase type I solution (CN), one wound was treated with 1 ml of 3 mg/ml collagenase I and covered by a collagen sponge (CN+Col), one wound was treated with 1 ml of 3 mg/ml collagenase I and covered by a collagen-PRP sponge (CN+Col+PRP).

Each wound was retreated every other day by adding new collagenase, collagen sponge, and collagen-PRP sponge, respectively. The wound healing results were monitored by determining the wound closure area. Tissue samples at wound area were harvested at day-12 post-surgery and treated with frozen section medium (Neg 50; Richard-Allan Scientific; Kalamazoo, MI) by the same protocols used for the sponge blocks and stored at −80°C for histological analysis.

### Histochemical and immunohistochemical analyses of tissue sections

The tissue block was cut into 10 μm thick sections and dried on glass slides at room temperature overnight. The sections were washed with PBS for 3 times, fixed with 4% paraformaldehyde for 30 min, and then washed with PBS for another 3 times. For histochemical analysis, the sections were stained with H&E. For immunohistochemical staining, the sections were incubated at room temperature in a humid chamber with 5% goat serum for 30 min. After the serum was carefully removed, the sections were incubated either with mouse anti-collagen type I or rabbit anti-collagen III or mouse anti-MMP-3 antibodies (1:350; Santa Cruz Biotechnology, Inc., Santa Cruz, CA) or mouse anti-von Willebrand Factor (vWF) (1:200; Bio-Rad, Raleigh, NC) antibodies at room temperature for 2 hrs. The tissue sections were washed with PBS 3 times, the expression of collagen I, MMP-3, and vWF were determined by Cy3-conjugated goat anti-mouse IgG (1:500; Santa Cruz Biotechnology) and collagen III expression was tested by Cy-3-conjugated goat anti-rabbit IgG (1:500; Santa Cruz Biotechnology) at room temperature for 1 hr, further washed with PBS 3 times, and Hoechst fluorochrome 33342 (1μg/ml; Sigma, St. Louis, MO) was used for staining the nuclei at room temperature for 5 min. Finally, the sections were washed with PBS three times and the stained sections were examined using fluorescence microscopy.

### Statistical analysis

Data are presented as mean plus and minus standard deviation (SD). For *in vitro* experiments, at least three replicates for each experimental condition were performed, and the presented results are representative of these replications. For *in vivo* experiments, 8 samples for each group were used for wound size evaluation at different time points and 3 slides from the surface, middle, and bottom of each wound area of the rat skin were histologically analyzed (total 24 slides for each group). Two-tailed student t-test was used for statistical analysis. Differences between two groups were considered significant when the *p*-value was less than 0.05.

## Abbreviations

CN: collagenase
Col: collagen
DRT: dermal regeneration template
ECM: extracellular matrix
HAc: acetic acid
MMPs: matrix metalloproteinases
NaOH: sodium hydroxide
PRP: platelet-rich plasma
SEM: scanning electron micrograph
TGF-β1: transforming growth factor beta 1
vWF: Von Willebrand factor
